# Prediction, attention, and unconscious processing in hierarchical auditory perception

**DOI:** 10.3389/fpsyg.2013.00955

**Published:** 2013-12-18

**Authors:** Sébastien Paquette, Geneviève Mignault Goulet, Kathrin Rothermich

**Affiliations:** ^1^International Laboratory for Brain Music and Sound Research, Department of Psychology, Center for Research on Brain, Language and Music, University of MontrealMontreal, QC, Canada; ^2^School of Communication Sciences and Disorders, Faculty of Medicine, McGill UniversityMontreal, QC, Canada

**Keywords:** predictive coding, prediction, attention, unconscious processing, amusia

In order to adjust to an ever-changing environment, our brain constantly constructs predictions for various inputs. The auditory system in particular has been shown to automatically use predictions to facilitate sequential processing. *Predictive coding theories* suppose that the brain extracts regularities to actively predict what is next. It is assumed that brain functions consist of a hierarchical model of predictions guided by *bayesian inference* (Friston, 2005); predictions coming from higher levels act as empirical priors for representations in lower levels, where sensory events are used to update the current predictions. In the end, the prediction model with the highest correctness probability determines the conscious percept.

Event-related potentials (ERPs) have been shown to reflect predictive activity in the brain. For instance, unexpected stimuli typically elicit an MMN component, irrespective of the participants' attention to the stimulus. The MMN is observed 100–250 ms following the onset of a deviant stimulus and is often interpreted as resulting from a mismatch between a memory record and the representation of the actual stimulus (Näätänen et al., 2007). The P300, on the other hand, is highly dependent on attention and usually elicited 300–500 ms after a stimulus onset (Picton, 1992; Schwartze et al., 2011); it has been interpreted as a surprise response to an infrequent stimulus (a disconfirmation of predictions; Wacongne et al., 2011). Though connections have been made between the MMN, the P300, and predictive coding theory (Wacongne et al., 2011), it remains unclear how they support the hierarchical organization of prediction.

Chennu et al. (2013) address this issue via a complex paradigm manipulating both attention and stimulus expectancies. The paradigm was developed to specifically investigate ERPs in response to prediction errors. The authors presented a set of auditory stimuli to participants while recording ERPs to unpredictable events (infrequent changes), the so-called *oddball paradigm*. They presented different sequences of five tones that were either all identical (standards) or had the last tone differ from the first four. All *local standards* were monaurally presented to either the right or the left ear, whereas *local deviants* were either presented in the opposite ear or were presented in the same ear with a pitch change. Moreover, the authors manipulated the order of the sequences on a more global scale, creating a higher hierarchical level of unpredictable events. While *local* deviants appear within a sequence (i.e., AAAA**B**), *global deviants* consisted of sequences manipulated in pitch or laterality that differed from the global standard (i.e., AAAAB AAAAB **AAAAA**). Additionally, the authors assigned participants to one of three conditions: (C1) where participants had to count all *uncommon sequences* (i.e., focus on the global pattern), (C2) where participants had to count all *deviant tones* (i.e., focus on local deviations), and (C3) where participants had to perform a demanding visual task (i.e., divert their attention).

Globally, the authors described the effects of attention and expectancy on error signal processing at different levels in the predictive hierarchy. The modulation of early auditory processes (MMN) was linked to the amount of prediction error: attentional precision (laterality vs. pitch deviant) enhanced early responses to prediction violations whereas top-down expectation (C2) reduced them. In contrast, the modulation of late auditory processes (P300) was highly dependent on attentional engagement (see also, van Zuijen et al., 2006), but also modulated by attentional precision (laterality vs. pitch deviant) and by top-down expectancies (C2). These results contribute directly to the functional differentiation of the MMN and the P300. As such, prediction errors at different levels within that hierarchy can be directly attributed to specific ERP components, providing a unified narrative that can be used to study failures of hierarchical prediction.

Although their narrative is instructive, further investigation regarding unusual MMN/P300 patterns in special populations could help to understand hierarchical auditory perception. For example, congenital amusics suffer from a pitch perception deficit that makes them unable to detect wrong notes and recognize familiar melodies. This neurodevelopmental disorder cannot be explained by hearing loss or a more general cognitive deficit. More precisely, amusics are unable to detect pitch changes that are smaller than a semitone, whereas controls do so reliably (Hyde and Peretz, 2004; Peretz et al., 2008). While their difficulties are mostly confined to pitch perception problems, several studies have investigated the level of consciousness in which those deficits take place.

Using an oddball paradigm, it was demonstrated that the amusic brain does not produce a normal P300 when presented with small pitch changes in tone sequences (Peretz et al., 2005; Mignault Goulet et al., 2012; Moreau et al., 2013) or melodic sequences (Peretz et al., 2009). This finding cannot be explained by a lack of attentional resources because pitch changes greater than a semitone are correctly detected by amusics and generate a normal P300. However, those same small pitch deviations (not consciously perceived by amusics) are accurately processed at a pre-attentive level, as indexed by a normal MMN response to the deviant tone. The amusic brain, like the typical brain, automatically creates predictions and responds to errors for fine-grained pitch changes at an early level of processing, but fails to do so at a later attention-dependent level. Their incapacity to consciously perceive this information, even if they deploy attention, presents a unique opportunity to study modulations of the two components without having to rely on the subjects' inherent attentional capacities.

Further investigations of these unusual MMN/P300 patterns in amusics may reveal how the underlying prediction errors interact and how they are used to update predictions in response to an ever-changing environment. For example, it would be interesting to investigate if the MMN in amusics is attenuated by explicit top-down, attention-dependent expectations [decreased response when attending single tones (C2) compared to attending complex patterns (C1); Chennu et al. (2013)], providing an example of an interaction between explicit and implicit processing. Since amusics represent an “unconscious although attentive” population, further studies could also help explain the neural architecture behind the generation of these predictive components and clarify why the amusic brain cannot implement a normal predictive coding scheme.

## References

[B1] Chennu S. Noreika V. Gueorguiev D. Blenkmann A. Kochen S. Ibáñez A. (2013). Expectation and attention in hierarchical auditory prediction. J. Neurosci. 33, 11194–11205 10.1523/JNEUROSCI.0114-13.2013 23825422 PMC3718380

[B2] Friston K. J. (2005). A theory of cortical responses. Philos. Trans. R. Soc. Lond. B Biol. Sci. 360, 815–836 10.1098/rstb.2005.1622 15937014 PMC1569488

[B3] Hyde K. Peretz I. (2004). Brains that are out of tune but in time. Psychol. Sci. 15, 356–360 10.1111/j.0956-7976.2004.00683.x 15102148

[B4] Mignault Goulet G. Moreau P. Robitaille N. Peretz I. (2012). Congenital amusia persists in the developing brain after daily music listening. PLoS ONE 7:e36860 10.1371/journal.pone.0036860 22606299 PMC3350472

[B5] Moreau P. Jolicoeur P. Peretz I. (2013). Pitch discrimination without awareness in congenital amusia: evidence from event-related potentials. Brain Cogn. 81, 337–344 10.1016/j.bandc.2013.01.004 23434917

[B6] Näätänen R. Paavilainen P. Rinne T. Alho K. (2007). The mismatch negativity (MMN) in basic research of central auditory processing: a review. Clin. Neurophysiol. 118, 2544–2590 10.1016/j.clinph.2007.04.026 17931964

[B7] Peretz I. Brattico E. Järvenpää M. Tervaniemi M. (2009). The amusic brain: in tune, out of key, and unaware. Brain 132, 1277–1286 10.1093/brain/awp055 19336462

[B8] Peretz I. Brattico E. Tervaniemi M. (2005). Abnormal electrical brain responses to pitch in congenital amusia. Ann. Neurol. 58, 478–482 10.1002/ana.20606 16130110

[B9] Peretz I. Gosselin N. Tillmann B. Cuddy L. L. Gagnon B. Trimmer C. G. (2008). On-line identification of congenital amusia. Music Percept. 25, 331–343 10.1525/MP.2008.25.4.33122509257

[B10] Picton T. W. (1992). The P300 wave of the human event-related potential. J. Clin. Neurophysiol. 9, 456–479 1464675 10.1097/00004691-199210000-00002

[B11] Schwartze M. Rothermich K. Schmidt-Kassow M. Kotz S. A. (2011). Temporal regularity effects on pre-attentive and attentive processing of deviance. Biol. Psychol. 87, 146–151 10.1016/j.biopsycho.2011.02.021 21382437

[B12] van Zuijen T. L. Simoens V. L. Paavilainen P. Näätänen R. Tervaniemi M. (2006). Implicit, intuitive, and explicit knowledge of abstract regularities in a sound sequence: an event-related brain potential study. J. Cogn. Neurosci. 18, 1292–1303 10.1162/jocn.2006.18.8.1292 16859415

[B13] Wacongne C. Labyt E. van Wassenhove V. Bekinschtein T. Naccache L. Dehaene S. (2011). Evidence for a hierarchy of predictions and prediction errors in human cortex. Proc. Natl. Acad. Sci. U.S.A. 108, 20754–20759 10.1073/pnas.1117807108 22147913 PMC3251061

